# Green cities and vector-borne diseases: emerging concerns and opportunities

**DOI:** 10.2807/1560-7917.ES.2024.29.10.2300548

**Published:** 2024-03-07

**Authors:** Florence Fournet, Frédéric Simard, Didier Fontenille

**Affiliations:** 1 MIVEGEC, University of Montpellier, IRD, CNRS, Montpellier, France

**Keywords:** Greening, urban areas, vector-borne diseases

## Abstract

Aligned with the Sustainable Development Goals, nature-based solutions such as urban greening e.g. public gardens, urban forests, parks and street trees, which aim to protect, sustainably manage or restore an ecosystem, have emerged as a promising tool for improving the health and well-being of an ever-increasing urban population. While urban greening efforts have undeniable benefits for human health and the biological communities inhabiting these green zones, disease vector populations may also be affected, possibly promoting greater pathogen transmission and the emergence of infectious diseases such as dengue, West Nile fever, malaria, leishmaniosis and tick-borne diseases. Evidence for the impact of urban green areas on vector-borne disease (VBD) transmission is scarce. Furthermore, because of vast disparities between cities, variation in green landscapes and differing scales of observation, findings are often contradictory; this calls for careful assessment of how urban greening affects VBD risk. Improved understanding of the effect of urban greening on VBDs would support planning, monitoring and management of green spaces in cities to sustainably mitigate VBD risks for surrounding urban populations.

## Background

Since 2007, more of the world’s population live in cities than in rural areas, and by 2022, 4.4 billion of the total 8.1 billion people reside in urban centres, a number which is expected to double by 2050 [1]. In Europe, more than 75% of the population lives in urban areas. Despite environmental, demographic and infrastructural diversity, cities worldwide face similar challenges, including high population density, low biodiversity, vulnerability to climate change and significant mobility, as well as acute health and social disparities. 

To meet these challenges, many cities on every continent are adopting nature-based solutions, defined as ‘actions aimed at protecting, conserving, restoring, sustainably using and managing natural or modified ecosystems’ [2]. Urban greening programmes such as planting trees and flowers, developing community gardens, greening buildings, developing parks and forests, and green and blue networks in the form of a combination of water bodies e.g. rivers and marshes, and green spaces e.g. agricultural areas, parks, wastelands, aim to create or preserve refuge areas for biodiversity and facilitate the circulation of species through ecological corridors [3].

Urban greening is consistent with a 'One Health' approach, the Sustainable Development Goal 11 ‘*Make cities inclusive, safe, resilient and sustainable*’ [4], and the Target 12 *Enhance Green Spaces and Urban Planning for Human Well-Being and Biodiversity* of the Biodiversity Plan for Life on Earth [5]. In addition, urban greening initiatives positively impact the health and well-being of residents, through effects on air pollution, soil quality, heat islands, ultraviolet (UV) exposure, ozone concentration and noise pollution [6,7]. However, the impact of these effects on the distribution, spread and behaviour of arthropod vectors and vertebrate hosts of vector-borne pathogens — and therefore the influence on vector-borne disease (VBD) transmission risk — has yet to receive special attention.

The effect of changing urban environments on insects such as bees, butterflies and beetles is increasingly backed by evidence [8-10]. Yet, the connection between urban greening initiatives and the presence of arthropods linked to potential health risks — like mosquitoes, sandflies and ticks — are often overlooked [11]. Furthermore, urban green and blue spaces may facilitate the emergence and the spread of vector-borne pathogens to humans in multiple ways, from local increases in vector and reservoir diversity and abundance to an upsurge in human exposure e.g. as more people engage in outdoor recreational activities. Hence, it is imperative to identify and quantify these potential impacts of urban greening and blueing on VBD, evaluate the probability of new health problems arising from vectors in diverse socio-ecological contexts and infectious patterns, and provide sustainable and acceptable measures for mitigation or adaptation.

In this Perspective article, we highlight emblematic examples of emerging VBD, drawn from around the world, that have affected vertebrates — particularly humans — and which we anticipate will be sensitive to urban greening and blueing in which cities are engaging to become more sustainable.

## Urban greening and blueing, mosquitoes and vector-borne disease transmission

Urban transmission of VBD such as dengue, chikungunya and Zika is driven by the introduction of the virus into the ecosystem – often through travellers who frequently transit cities – and the abundance and behaviour of urban-adapted vectors, *Aedes albopictus* and *Ae. aegypti* mosquitoes. Surprisingly, the connection between urban greenery, vector density and arboviral health risk in humans remains scant, and conflicting patterns have been reported. For example, while a dengue outbreak in Tokyo, Japan in 2014 was due to *Ae. albopictus* thriving in the city’s urban parks [12], Sao Paulo, Brazil experienced in 2010–11 lower dengue incidence in vegetated city pockets where cooler temperatures prevailed — though whether the reduction in transmission was solely due to vegetation or other socioeconomic factors is debatable [13]. Research in functional ecology, including ecological network approaches [14], should improve our understanding of the intimate relationships between environmental factors e.g. host plant diversity and phenology, vegetation structure, water management practices, and characteristics of the vector e.g. the biology of both larvae and adult *Aedes* mosquitoes in urbanised areas.

Other mosquito species, such as those in the *Anopheles gambiae* complex which are major vectors of malaria in Africa, have acclimatised to urban surroundings by colonising breeding sites in urban agriculture zones, as demonstrated by Afrane et al. [15] in Kumasi, Ghana and Dongus et al. [16] in Dar-es-Salam, Tanzania. Meanwhile, *Anopheles stephensi*, an Asian mosquito gradually infiltrating African cities because of trade and human movement, does not rely as much on vegetation for breeding as other *Anopheles* spp. This vector rather populates breeding spots tied to human activities, notably water storage containers, prevalent in cities with inadequate water management [17].

## Biodiversity and the emergence of zoonotic vector-borne diseases

Urbanisation is known to reduce biodiversity through habitat loss and fragmentation. Therefore, green spaces can benefit urban ecosystems by enhancing biodiversity through the creation of refuge areas for animals and plants, but also by fostering their movement along green networks. Shifts in the diversity of birds and vectors within urban environments can significantly influence the prevalence of vector-borne diseases and the potential for disease spillover.

*Culex* mosquitoes are the principal vectors of West Nile virus (WNV) and Usutu virus in birds. The risk for spillover transmission of these viruses to humans varies in complex ways with culicid and avian species richness. For example, in the summer of 1999 with high temperatures in New York City, United States (US), surges of urban *Culex pipiens* and birds, which were partly associated with urban vegetation, fuelled a WNV outbreak in humans [18]. In contrast to the WNV outbreak in New York, a study conducted in 2010–12 in the major urban centre of Atlanta, US, revealed no increase in human WNV cases despite high virus prevalence in both mosquito vectors and avian hosts [19]. Interestingly, in intra-urban forest islands, hosts highly susceptible to WNV such as the American robin were edged out by less susceptible hosts, like the red cardinal, during peak months of virus transmission in August and September, reducing the risk of spillover to humans. Although urbanisation has reduced overall species diversity among birds, some species have become widely established, while others have also adapted to the urban environment. Among the bird species present in cities in both the US and in Europe, many are competent hosts for WNV.

Hence, enhancing urban biodiversity by greening may affect not only arthropod vector diversity and abundance but also reservoir hosts and the infectious agents themselves. An example observed near Madrid, Spain, between 2009 and 2012, when sizeable populations of sandflies (*Phlebotomus perniciosus*) and hares (*Lepus granatensis*), prevalent in urban parks, caused the emergence of 446 cases of visceral and cutaneous leishmaniasis in humans [20]. Moreover, linking urban parks and forests via ecological corridors and green-blue networks may facilitate the introduction and establishment of ticks and their vertebrate hosts, particularly mammals, entwined in urban cycles of tick-borne diseases such as encephalitis, haemorrhagic fevers and borrelioses [21]. In 2017, on Staten Island, New York, US, park connectivity and tree density influenced the density of *Ixodes scapularis* tick populations and their infection by *Borrelia burgdorferi*, which causes Lyme disease [22]. This pattern also holds in Europe, with an uptick in Lyme borreliosis and tick-borne encephalitis cases noted in green urban and peri-urban zones since 2010 [23,24]. Efforts to increase urban biodiversity can therefore favour the presence of vectors or hosts for infectious pathogens, which can lead to the introduction of VBD such as West Nile fever or tick-borne diseases.

## Urban green and blue areas: a tool to mitigate vector-borne disease risk in cities

While urban green areas may create cooler ecological niches, this will likely increase the genetic and functional diversity of both the pathogens and their vectors. This heightened diversity is not necessarily unfavourable because it does not inevitably translate into heightened VBD transmission risk. Indeed, a 'dilution effect' may mitigate health risks if green zones also drive an increase in the abundance of poor and non-competent hosts, which act as dead ends for pathogens or divert vectors from biting human hosts [25]. The size, composition, structure, distribution and interconnectivity of green spaces all influence habitat suitability for vectors but also competitors and predators, and thus vector capacity and pathogen transmission [14]. A better understanding of how the greening of cities affects the web of interactions underlying VBD transmission may allow for more thoughtful engineering of city greening, with nature-based solutions emerging as a powerful tool for sustainable management of vectors and VBD-emergence and transmission risk in cities.

This means that research must provide compelling evidence regarding the impact of vegetation on vector-borne diseases risk, using rigorous methodologies such as quasi-experimental approaches [26]. Furthermore, these efforts must rely on approaches that include all relevant stakeholders from different disciplines, sectors and decision-making levels throughout the process, in order to identify user needs and be able to meet them ultimately [27]. To understand the impact of green spaces on the risk of vector-borne diseases, supporting data on their role in vector ecology are needed, including their ability to heighten the risk of vector-borne diseases through facilitating interactions among humans, animals, and vectors (Box).

Box **Key questions relating to urban green spaces and vector-borne diseases**

• Is the impact on vectors the same, independent of the type of green space e.g. forest, park, green wall, green roof?

• To what extent do green spaces amplify the abundance and diversity of vectors?

• What protocols can be designed for entomological and environmental data monitoring, encompassing variables such as temperature, relative humidity and landscape metrics?

• What indicators can be used to define and assess the risk of vector-borne diseases?

## Conclusion

Nature-based solutions have been recognised by national, European and global policies for their potential to meet global biodiversity targets and deliver multiple benefits to society, including in cities, where a large part of the global population is now concentrated. Europe is engaged in various strategies to tackle global environmental and climate challenges such as the Green Deal and the Biodiversity Strategy 2030 [28,29]. The importance of systematically integrating green spaces and nature-based solutions into urban planning to promote healthy ecosystems is emphasised. Positive effects linked to urban greening have been reported on human health regarding respiratory diseases, cardiovascular diseases and mental health. Greening cities, however, could also be conducive to the emergence of VBD. To ensure the viability of current and future urban greening initiatives, it is necessary to proactively identify and monitor how greening affects the risk of VBD, and to anticipate nature-based management solutions for tomorrow's cities. Without action, the possible negative ecological, economic and social effects of an increase in urban VBD could jeopardise the acceleration and extension of nature-based solutions in Europe as promoted by the Green Deal.
